# Self-care interventions for sexual and reproductive health in humanitarian and fragile settings: a scoping review

**DOI:** 10.1186/s12913-022-07916-4

**Published:** 2022-06-07

**Authors:** Angela Dawson, Hannah Tappis, Nguyen Toan Tran

**Affiliations:** 1 Australian Centre for Public and Population Health Research, Faculty of Health, University of Technology Sydney, PO Box 123, Sydney, NSW 2007 Australia; 2 Jhpiego, 1615 Thames St, Baltimore, MD USA; 3 Johns Hopkins Center for Humanitarian Health, 615 N. Wolfe St, Baltimore, MD USA; 4 Faculty of Medicine, University of Geneva, Rue Michel Servet 1, 1211 Geneva 4, Switzerland

**Keywords:** Self-care, Reproductive health, Sexual health, Humanitarian health

## Abstract

**Background:**

Self-care is the ability of individuals, families, and communities to promote health, prevent disease, maintain health, and manage illness and disability with or without a health care provider. In resource-constrained settings with disrupted sexual and reproductive health (SRH) service coverage and access, SRH self-care could play a critical role. Despite SRH conditions being among the leading causes of mortality and morbidity among women of reproductive age in humanitarian and fragile settings, there are currently no reviews of self-care interventions in these contexts to guide policy and practice.

**Methods:**

We undertook a scoping review to identify the design, implementation, and outcomes of self-care interventions for SRH in humanitarian and fragile settings. We defined settings of interest as locations with appeals for international humanitarian assistance or identified as fragile and conflict-affected situations by the World Bank. SRH self-care interventions were described according to those aligned with the Minimum Initial Services Package for Reproductive Health in Crises. We searched six databases for records using keywords guided by the PRISMA statement. The findings of each included paper were analysed using an a priori framework to identify information concerning effectiveness, acceptability and feasibility of the self-care intervention, places where self-care interventions were accessed and factors relating to the environment that enabled the delivery and uptake of the interventions.

**Results:**

We identified 25 publications on SRH self-care implemented in humanitarian and fragile settings including ten publications on maternal and newborn health, nine on HIV/STI interventions, two on contraception, two on safe abortion care, one on gender-based violence, and one on health service provider perspectives on multiple interventions. Overall, the findings show that well-supported self-care interventions have the potential to increase access to quality SRH for crisis-affected communities. However, descriptions of interventions, study settings, and factors impacting implementation offer limited insight into how practical considerations for SRH self-care interventions differ in stable, fragile, and crisis-affected settings.

**Conclusion:**

It is time to invest in self-care implementation research in humanitarian settings to inform policies and practices that are adapted to the needs of crisis-affected communities and tailored to the specific health system challenges encountered in such contexts.

**Supplementary Information:**

The online version contains supplementary material available at 10.1186/s12913-022-07916-4.

## Background

The World Health Organization (WHO) defines self-care as the ability of individuals, families, and communities to promote health, prevent disease, maintain health, and cope with illness and disability with or without a health care provider [1]. Sexual and reproductive health (SRH) self-care information and skills passed down through centuries have enabled generations of women and girls to manage their menses, fertility, pregnancy, childbirth, and care for their infants and children [2]. In resource-constrained settings with limited or disrupted SRH service coverage and access, such as humanitarian crises, SRH self-care could play a critical role [3]. However, for self-care to be an integral and sustainable component of health systems, it should be grounded in a people-centred, gender-sensitive, and rights-based framework [4, 5].

The WHO conceptual framework for self-care interventions is centred on these principles. It reflects the notion that programmatic considerations for introducing or supporting self-care interventions will vary depending on the setting, intervention time, where and how they are accessed, and the health system links required to support care [6]. With this in mind, the WHO “living guidelines” include a best practice statement recognizing that the provision of tailored and timely support for self-care interventions in humanitarian settings should form part of emergency preparedness plans and be provided as part of ongoing responses [7]. However, no details are provided on conditions that must be met or how to tailor support to ensure an enabling environment for people to safely and effectively adopt SRH self-care practices where armed conflicts, extreme weather events, or other crises disrupt health systems.

Recently published studies reviewed and synthesized the literature on the effectiveness of different components of SRH self-care and, to some extent, feasibility and acceptability. For example, Kennedy et al. identified a limited body of literature showing that women who acquire oral contraceptives over-the-counter may have higher continuation retention rates and limited contraindicated use, with the over-the-counter availability of oral contraceptives overwhelmingly supported by patients and physicians [8]. Another review suggests that the self-administration of depot medroxyprogesterone acetate subcutaneous injectable contraception (DMPA-SC) can equal or enhance contraceptive continuation rates compared with provider administration while causing no substantial increase in pregnancy or safety issues [9]. Yeh et al. identified a growing body of research suggesting that human papillomavirus (HPV) self-sampling can boost cervical cancer screening uptake with only a minor effect on linkage to clinical assessment and treatment compared to standard of care [10]. Ogale et al. reviewed the effectiveness of self-collected sexually transmitted infection (STIs) samples [11]. The meta-analysis showed an increase in the overall uptake of STI testing services by three folds and case finding by two folds.

Except for the DMPA-SC review, which identified research done in Malawi, Senegal, and Uganda, the other reviews found studies originating primarily from high-income countries. Although SRH conditions are among the leading causes of mortality and morbidity among women of reproductive age globally, with 61% of maternal deaths occurring in countries facing fragility and crisis, none of the studies were in humanitarian settings [12]. Our research aimed to fill this knowledge gap by reviewing and synthesizing the evidence on SRH self-care interventions in humanitarian and fragile settings.

## Methods

We undertook a scoping review to identify the design, implementation, and outcomes of self-care interventions for SRH in humanitarian and fragile settings. We aimed to describe the types of SRH self-care interventions studied, and findings related to the factors that determine the implementation and uptake of self-care interventions, the barriers to the use of self-care interventions, and the experience of consumers and health professionals concerning the use of self-care interventions for SRH in crisis contexts.

We employed the five-stage process for conducting a scoping review described by Levac et al. [13]. This comprised the development of the research question, identifying pertinent studies, articulating the selection criteria, mapping the data, and reporting the results. This scoping review is registered as a project on the Open Science Framework (https://osf.io/wq7sy/).

### Search strategy and eligibility criteria

We developed an inclusion criterion based on definitions of fragile settings as countries included on World Bank Group’s list of fragile and conflict-affected situations in FY21 [14] and humanitarian settings as those countries or regions that had a Humanitarian Response Plan (*HRP*) prepared in response to a protracted or sudden onset emergency that required international humanitarian assistance [15]. We developed a list of countries and identified the HRPs, emergencies, and dates of these to guide our inclusion and exclusion criteria (see Additional file 1). We defined SRH self-care interventions as those either described as such by authors or included in Tran et al.’s list of SRH self-care interventions that are aligned with the Minimum Initial Services Package for Reproductive Health in Humanitarian Crises (MISP) [16]. Extensive scoping searches were undertaken to define the inclusion criteria and search terms as few studies described the location in which their research took place as a “fragile setting” or “humanitarian crisis”, nor did they always refer to their intervention of focus as “self-care.”

**Fig. 1:**
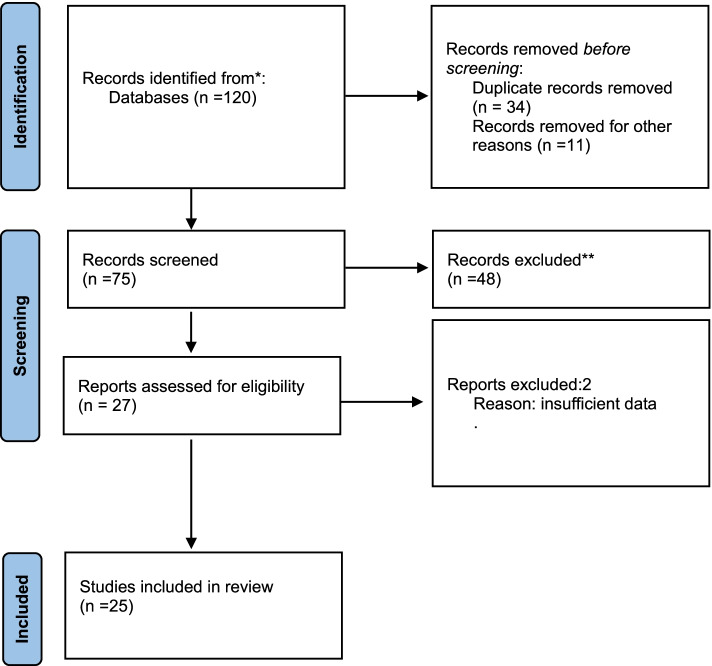
Literature Search

We searched six databases (Embase, Maternity and Infant Care, CINAL, Web of Science, PubMed, and Medline) for records from 2000 using defined keywords. We included peer-reviewed primary research papers and conference abstracts and excluded discursive papers. As self-care in crisis is an emerging field, we included conference abstracts to provide insight into peer-reviewed preliminary research beyond full articles. Studies have found little difference between conference abstracts and fully published papers of the same study findings [17]. Figure 1 outlines the search processes as per the PRISMA statement [18] and Additional file 2 provides details of the databases, search terms and numbers of documents returned, screened and included. The PRISMA Extension for Scoping Reviews (PRISMA-ScR): A checklist was used to guide the reporting of this study (see Additional file 3) [19].

### Study selection and quality assessment

Retrieved publications were imported into the online software product Covidence and screened by the three authors using the inclusion criteria. We used the Critical Appraisal Skills Programme checklist to appraise qualitative studies and randomised control trials [20]. Quantitative studies were assessed using a variation of the Newcastle-Ottawa Scale assessment tool [21], and the Appraisal Tool for Cross-Sectional Studies [22]. The Mixed Methods Appraisal Tool was used to assess mixed-method studies [23]. Quality assessment was undertaken by one author (AD) and independently verified by the other authors so agreement was reached. No studies were excluded.

### Data extraction and analysis

The characteristics of included studies were first plotted according to the country context, crisis setting, self-care intervention, method aim, and relevant findings. We undertook a content analysis of the findings of each included paper using an a priori framework to identify information concerning effectiveness, acceptability and feasibility of the self-care intervention, places where self-care interventions were accessed and factors relating to the environment that enabled the delivery uptake of the intervention. Data were then extracted from the results sections of included studies according to these variables of interest. Coding of relevant extracted data from the documents was independently undertaken by AD and HT and NTT, and consensus was reached through discussion where there was disagreement. Findings are presented by type of SRH intervention.

## Findings

### Characteristics of included papers

We identified 25 publications on SRH self-care implemented in humanitarian contexts in more than ten countries. Table 1 shows the characteristics of the 25 included publications, which comprise 10 publications on maternal and newborn health (MNH) interventions and practices, nine on HIV/STI interventions, two on contraception, two on safe abortion care, one on gender-based violence (GBV), and one on multiple SRH interventions. Although all met location-related inclusion criteria, only ten (40%) of the publications described the study setting as humanitarian crisis or conflict-affected.

**Table 1:** Summary of included studies

Reference	Country context	Type of crisis	Self-care intervention	Method	Study population	Aim	Relevant findings
(Abbas, Mirzazada et al. 2020) [24]	6 districts in Badakhshan Province, Afghanistan	Conflict	Misoprostol for treatment of PPH	Double-blind, randomized placebo-controlled trial	2337 women, of whom 1884 delivered at home & 453 delivered at a health facility	To assess the effectiveness, safety, & acceptability of misoprostol use to treat PPH after its use prophylactically	Among the 1884 women who delivered at home, 98.7% reported self-use of misoprostol for PPH prevention. 4.4%, 82/1884 were diagnosed with PPH & administered treatment.
(Adejuyigbe, Bee et al. 2015) [25]	2 LGAs in Borno State in North East Nigeria	Conflict	Essential newborn care (thermal care	Qualitative newborn care narratives, observations of bathing & in-depth interviews	10 Bura & 10 Kanuri recent mothers, grandmothers, fathers, health workers & birth attendants.	To explore thermal care practices.	Near universal early bathing of babies, link between delayed bathing & body odour later in life. Skin to skin care was not practised.
(Ammerdorffer, Laws et al. 2021) [26]	Lebanon	Conflict	Self-administration of injectable contraception, OTC oral contraceptive pills, self-management of medical abortion process in the first trimester.	Document review	Lebanon National Drug Index, Lebanon National Drugs Database, Ministry of Public Health website	To review the regulation status of self-administration of injectable contraception, OTC oral contraception & self-management of medical abortion	No official procedure to change Prescription only medicine (POM) to over-the-counter (OTC). Ulipristal acetate is only available OTC not provided by Ministry of Health but is available at pharmacies to purchase.
(Berry-Bibee, St Jean et al. 2018) [27]	Cap Haitien, Haiti	Natural disaster	Self-management of medical abortion process in the first trimester.	Mixed methods: 8 focus groups with women & interviews with women’s health providers, survey to pregnant or recently pregnant women	62 women, 13 health providers, 255 pregnant or recently pregnant women < 20 weeks gestation presenting to the hospital.	To document access to illegal abortion, unscheduled hospital visits related to unsafe abortion methods & perceived barriers to abortion-related care.	78 of 150 women who stated that their pregnancy was unintended reported attempting an induced abortion using misoprostol either alone or as a part of the medication/herb regimen for their self-managed abortion (85.1%, *n* = 63).
(Bertrand, Bidashimwa et al. 2018) [28]	Kinshasa, Democratic Republic of the Congo	Conflict	DMPA-SC self-injection.	Prospective cohort: Women who selected DMPA-SC interviewed upon acceptance (baseline) & then 3, 6 & 12 months later.	850 women selected DMPA-SC at a community outreach event beside a health centre.	To assess the acceptability & feasibility of DMPA-SC self-injection.	640 (75.3%) opted for self-injection over being injected by the nursing students for reasons of convenience & personal agency. 47.5% were anxious at baseline (for fear of needles or injecting incorrectly). Over 80% reported feeling very ready after training, confident that they knew how to self-inject & confident that they would remember the next injection date. At 3 months, 97% described it as easy. Half (54%) experienced side effects, mainly menstrual irregularities, the main reason for discontinuation. At 6-month follow-up, self-injectors cited effectiveness & ease of use, one quarter reported side effects.
(Burke 2018) [29] Conference abstract	Northern Haiti	Natural disaster	Misoprostol for prevention of PPH.	Participants received group & individual counselling on birth preparedness, PPH prevention, & importance of facility birth. At enrolment, participants received 3200 mcg tablets of misoprostol & instructions for self-administration after birth. A postpartum questionnaire was administered 2 weeks after birth.	338 women 32 weeks gestation.	To explore the feasibility of advanced distribution of misoprostol to pregnant women to self-administer at the time of delivery to prevent PPH.	72% of participants delivered in a health facility & 80% of all participants took misoprostol. Among women who delivered at home, 87% took misoprostol & all took the medication correctly after delivery.
(Conserve, Michel et al. 2020) [30]	Haiti	Natural disaster	Self-testing HIV (HIVST)	16 key informant interviews & 9 focus groups.	44 healthcare workers, 31 Option B+ clients, &13 men, representatives of the Ministry of Health & a NGO involved in HIV partner services.	To assess the perspectives of women living with HIV (WLWH), their male partners, & healthcare professionals on the perceived advantages & disadvantages of HIVST, & recommendations for implementing HIVST.	HIVST advantages included an increase in the number of people who would learn their HIV status & start treatment. Perceived disadvantages were lack of support to ensure self-testers initiate treatment, uncertainty about male partner’s reaction, risk of violence towards women delivering HIVST kits after receiving an HIVST kit from a woman, & the inability of women to counsel a man in case his self-test result is positive. Need to coupling HIVST distribution with public information, education & communication through media & social marketing, relying on community health workers to mediate use of HIVST & ensure linkage to care.
(de Vries, Hamad et al. 2021) [31]	Gaza	Conflict	Essential new-born care	Mixed methods: qualitative informant interviews, in-depth interviews &/or focus group discussions secondary analysis of Ministry of Health annual reports, central statistics & MICSs.	Women targeted by the programme, non-targeted women, husbands, & home visitors.	To evaluate a postnatal home visiting program.	Women in the program demonstrated improved breastfeeding practices & increased uptake of breastfeeding, reduced harmful traditional norms & practices, improved relationships between women & providers & women’s self-esteem.
(Draiko, McKague et al. 2021) [32]	Jubek County, South Sudan	Conflict	Essential new-born care	Pre/post quasi-experimental study.	3143 pregnant women from 6 rural communities: 1825 women in the treatment group & 1318 women in the control group.	To assess the effectiveness of applying chlorhexidine gel to the umbilical cord stump on cord sepsis & neonatal mortality rates.	The neonatal cord infection rate among the treatment group was 17.0%, compared to 38.9% in the control group (*P* < 0.05). Neonatal mortality was least in the intervention (1.3%) & highest in the control (13.3%) group.
(Gill and Tam 2021) [33] Conference abstract	Venezuela	Complex	Self-management of medical abortion process in the first trimester	Mixed-methods implementation research study co-design Qualitative interviews & survey.	Women, grassroots community-based organizations, healthcare providers, SRH advocates & program experts.	Understand how Venezuelan women access SRH information & services; preferences for design of a digital tool to safely facilitate self-managed abortions & contraception access.	30% of surveyed Venezuelan women had an abortion, half used unsafe methods. 83% own a smartphone, 77% have internet access. Women were supportive of a digital tool.
(Haver, Ansari et al. 2016) [34]	20 districts, across 5 provinces (Faryab, Jawzjan, Kabul, Badakhshan, & Bamyan) Afghanistan	Conflict	Misoprostol for prevention of PPH	Cross-sectional pre & post household survey.	Pre-intervention households- (*n* = 408) & post intervention households (*n* = 408).	To determine the effectiveness of advance distribution of misoprostol for self-administration.	Uterotonic use among women in the sample increased from 50.3% preintervention to 74.3% postintervention. Significant difference in uterotonic use at home births was observed among women who lived farthest from a health facility (> 90 min self-reported travel time) compared to women who lived closer (88.5% vs 38.9%; *P* < 0001). All women who accepted misoprostol & gave birth at home used the drug. No maternal deaths were identified among those women who used misoprostol.
(Komakech, Lubogo et al. 2020) [35]	Refugee camps Adjumani district, west Nile, Uganda	Conflict	Essential new-born care	Cross-sectional semi-structured questionnaire.	561 mothers of infants aged 0–6 months.	To assess essential newborn care practices & its determinants.	57% of the mothers breastfed their newborns within one hour. 50.1% of mothers cleaned the umbilical cord of their newborns. 17% of the newborns received optimal thermal care immediately after birth. Mothers aged 20–24 years (OR 0.38, CI 0.17–0.96) & those involved in subsistence farming (OR 0.67, CI 0.38–1.45) were less likely to practice good newborn care compared to those in other occupations.
(Lathrop, Burke et al. 2018) [36] Conference abstract	Northeast Department of Haiti	Natural disaster	Misoprostol for prevention of PPH	12 semi-structured focus groups.	Health providers, community health workers (CHWs), traditional birth attendants (TBAs) & community leaders.	To explore community acceptability of advanced distribution of misoprostol to pregnant women.	The TBAs & CHWs felt their participation in the misoprostol strengthened pregnant women’s trust in their work. The majority felt that women had positive experiences with the drug. Several providers said the misoprostol program strengthened the collaboration between health centres, CHWs, TBAs & community leaders. They felt this was based on understanding that the program was life-saving & brought positive health outcomes to their communities. Sustainability of the intervention was important to several community leaders. All participants felt the intervention was acceptable to their communities.
(Logie, Abela et al. 2021) [37]	Eastern Mediterranean Region	Conflict	All SRH self-care	Online cross-sectional Global Values & Preferences Survey (open questions only to be extracted survey results cannot be disaggregated)	Participants from crisis setting included Afghanistan (2), Lebanon (4), Somalia (1), Sudan (3), Syria (1), Yemen (1).	To access, knowledge, perceived challenges, & recommendations for the future.	Benefits acknowledged, need for linked care via hot lines, apps, home visits & transport to facility, provider training on interventions & dealing with self-effects & complications, issues with stigma in pharmacies noted in Syria.
(Maatouk, El Nakib et al. 2021) [38]	Lebanon	Conflict	HIV self-testing	Audit of National AIDS Program data	Men who have sex with men who received a HIVST kit.	To describe the effectiveness of implementing HIVST.	NGOs distributed 1103/1380 (79.9%) HIVST kits to their beneficiaries. The NGOs collected feedback on 111/1103 kit results, of which two were HIV-positive. From Jan-May 2020, 625/780 HIVST kits (80.1%) were distributed. This period was divided into pre-COVID-19 & during COVID-19. The follow-up with the beneficiaries during COVID-19 was much improved because of the absence of on-site activities, shifting more efforts towards HIVST (449/625).
(O’Laughlin, He et al. 2018) [39]	Nakivale refugee settlement, southwestern Uganda	Conflict	HIV self-testing	Household survey	566 adults living in 319 homes were visited.	To determine the feasibility & acceptability of home-based HIV testing.	90% of eligible adults noted to be living in those households at 3 visits, confirming this approach is feasible. 75% of eligible adults encountered participated in HIV testing & received their results, reflecting the acceptability of home-based testing.
(Sanghvi, Ansari et al. 2010) [40]	Rural Afghanistan	Conflict	Misoprostol for prevention of PPH	Nonrandomized experimental control design	3187 women, 2039 in the intervention group & 1148 in the control group.	To test the safety, acceptability, feasibility, & effectiveness of community-based education & distribution of misoprostol for prevention of PPH.	Of the 1421 women in the intervention group who took misoprostol, 100% correctly took it after birth, including 20 women with twin pregnancies. Adverse effect rates were lower in the intervention group than in the comparison group. Among women in the intervention group, 92% said they would use misoprostol in their next pregnancy. In the intervention area where community-based distribution of misoprostol was introduced, near-universal uterotonic coverage (92%) was achieved compared with 25% coverage in the control areas.
(Sibanda, d’Elbee et al. 2019) [41]	rural Zimbabwe	Complex crisis	HIV self-testing	Community cluster randomised control trial.	40 village groups.	To compare a community-led versus an established paid distribution (PD) community-based HIVST model.	From Oct 2018 to August 2019, 27,812 & 36,699 HIVST kits were distributed in community-led & PD communities, respectively. 11,150 participants & 5683 were surveyed in community-led arm. New HIV diagnosis was reported by 211 (3.7%) community-led versus 197 (3.6%) PD arm participants, adjusted OR (aOR) 1.1 (95% CI 0.72 to 1.56); 318 (25.9%) community-led arm participants linked to post-test services versus 361 (23.9%) in PD arm, aOR 1.1 (95% CI 0.75 to 1.49. Cost per HIVST kit distributed was US$6.29 & US$10.25 for PD & community-led HIVST, both lower than 2016/2017 costs for newly implemented PD (US$14.52).
(Smith, Dimiti et al. 2014) [42]	Mundri East County in Western Equatoria State, South Sudan	Conflict	Misoprostol for prevention of PPH	Descriptive observational study involving audit of facility registers, surveys of providers & women postpartum.	Women at approximately 32 weeks, 135 female home health promoters, Prenatal-care provider	To evaluate the program performance in terms of uterotonic coverage for facility & home births at the population level, & serious adverse effects of misoprostol use, effectiveness of counselling & program acceptability.	533 home births & 394 facility-based births were reported. Misoprostol was distributed in advance to 787 (84.9%) pregnant women, of whom 652 (82.8%) received the drug during home visits. Among the women who delivered at home, 527 (98.9%) reported taking misoprostol. A uterotonic for PPH prevention was provided at 342 (86.8%) facility-based deliveries. Total uterotonic coverage was 93.7%. No adverse events were reported.
(Tol, Leku et al. 2020) [43]	rural refugee settlements in northern Uganda	Conflict	Positive coping methods	cluster randomised trial.	694 female South Sudanese refugees	To assess the effectiveness of a facilitator-guided, group-based, self-help intervention.	Larger improvements for Self-Help Plus on psychological distress 3 months post intervention (β −1・20, 95% CI −2・33 to −0・08; *p* = 0・04; d − 0・26). Larger improvements for Self-Help Plus 3 months post-intervention for five of eight secondary outcomes (effect size range − 0・30 to − 0・36). Refugees with different trauma exposure, length of time in settlements, & initial psychological distress benefited similarly.
(Tonen-Wolyec, Batina-Agasa et al. 2019) [44]	Kisangani & Bunia Democratic Republic of the Congo	Conflict	HIV self-testing	Multicentre cross-sectional study	208 female sex workers (FSWs), 132 non FSWs.	To evaluate participants ability to read & interpret the results of a prototype HIVST.	2704 standardized tests (1248 positive, 1040 negative, 416 invalid) were interpreted; 2435 (90.1%) were correctly interpreted, 269 (9.9%) were misinterpreted. In FSWs & non-FSWs, test results were correctly interpreted in 87.4% (864/988) & 91.6% (1571/1716), respectively. Educational levels associated with the interpretation of positive, negative, & invalid HIV self-test results, but not “commercial sex work” & “language chosen for instructions for use.” Incorrect interpretation was significantly higher in participants with insufficient educational level than in those with sufficient education level for positive (13.1% vs 2.6%; adjusted OR: 4.5), negative (22.3% vs 2.6%; adjusted OR: 5.3), & invalid test results (23.8% v 6.4%; adjusted OR: 3.6).
(Tonen-Wolyec, Batina-Agasa et al. 2019) [45]	Kisangani, Democratic Republic of the Congo	Conflict	HIV self-testing	Cross-sectional, door-to-door survey.	628 adolescents	To evaluate the acceptability, feasibility, & accuracy of home-based, supervised HIVST.	Acceptability of HIVST was high (95.1%); 96.1% of participants correctly used the self-test, & 65.2% asked for verbal instructions. The majority of adolescents (93.5%) correctly interpreted their self-test results. Correct interpretation decreased significantly when adolescents had no formal education or attended primary school. The sensitivity of the Exacto Test HIV Self-test was estimated at 100%, while its specificity was 96.0%. The majority of participants (68.0%) affirmed that post-test counselling was essential, & that face-to-face counselling (78.9%) was preferred.
(Tonen-Wolyec, Kayembe Tshilumba et al. 2020) [46]	Kisangani, Democratic Republic of the Congo	Conflict	HIV self-testing	Randomized (1:1), non-blinded, non-inferiority trial using a blood-based & facility-based HIVST method.	530, adults 18 & 49 yrs., at high risk of acquiring HIV infection, did not know their HIV status, lived or worked in Kisangani for at least 6 months.	To compare the practicability & effectiveness of the two delivery approaches for HIVST, unassisted HIVST (UH) & directly assisted HIVST (DAH).	The rate of successfully performing the test was the same (93.2%) in the UH & DAH arms. The rate of correctly interpreting the results was 86.9% in the UH arm versus 93.2% in the DAH arm, for a difference of −6.3%. After the follow-up 72 h later, participants in the UH arm had a significantly lower chance of correctly interpreting the test results than those in the DAH arm (aRR: 0.60; *P* = 0.019). The positivity rate was 3.4% among the participants in the DAH arm & 1.7% among those in the UH arm, no significant differences were found between the two arms in the positivity rate, requests for assistance, & linkage to care. Willingness to buy an HIV self-test was higher in the UH arm than in the DAH arm (92.3% versus 74.1%; aRR: 4.20; *P* < 0.001).
(Tonen-Wolyec, Mbopi-Keou et al. 2019) [47]	Bunia, Democratic Republic of the Congo	Conflict	HIV self-testing	A representative cross-sectional study using a self-administered semi-structured questionnaire.	1012 university students	To describe the acceptability of HIVST in students.	Acceptability of unsupervised HIVST was higher in the group of young students as compared with older students & was markedly associated with prior knowledge on HIVST.
(Tonen-Wolyec, Mboumba Bouassa et al. 2020) [48]	Kisangani, Democratic Republic of the Congo	Conflict	HIV self-testing	Representative cross-sectional study using random sampling in clusters at three levels.	597 adolescents aged 15–19 years.	To determine the sociodemographic factors associated with adolescents preferring home-based HIVST over facility-based voluntary counselling and testing (VCT).	The majority of participants (323/597; 54.1% [95% CI: 50.0–58.0]) preferred home-based HIVST over facility-based VCT. In a multivariate analysis, male gender (adjusted OR: 1.5, 95% CI: 1.1–2.1), marriage or partnered civil status (adjusted OR: 1.8, 95% CI: 1.1–3.1), & previous knowledge about HIVST (adjusted OR: 4.2, 95% CI: 2.6–6.8) were associated with preference for homebased HIVST over facility-based VCT.

Table 2 summarizes the study outcomes of interest, as well as place(s) of access for self-care information and components of the ecosystem and enabling environment that enabled uptake of interventions.

**Table 2:** Summary of study findings

Outcomes of Interest			Place(s) of Access						Enabling Environment & Ecosystem							
Effectiveness (*n* = 11)	Feasibility (*n* = 12)	Acceptability (*n* = 14)	House-hold (*n* = 13)	Informal community care or organizations (*n* = 0)	Over the counter (*n* = 2)	Formal community-based or outreach services (*n* = 3)	Facility based (*n* = 2)	Peer/ Person to person (*n* = 2)	Governance & regulatory alignment (*n* = 1)	Commodity security (*n* = 1)	Health worker awareness, engagement & motivation (*n* = 16)	Community participation (*n* = 14)	Digital technology & platforms (*n* = 4)	Mass media/ educational materials (*n* = 8)	Information systems (*n* = 1)	Financing (*n* = 0)
**Self-care for mothers and newborns**																
(Abbas, Mirzazada et al. 2020) [24]																
✓	✓	✓	✓								✓					
(Adejuyigbe, Bee et al. 2015) [25]																
		✓	✓								✓	✓				
(Burke 2018) [29]																
	✓										✓	✓				
(Haver, Ansari et al. 2016) [34]																
✓			✓								✓	✓				
(Lathrop, Burke et al. 2018) [36]																
		✓									✓	✓				
(Sanghvi, Ansari et al. 2010) [40]																
✓	✓	✓	✓			✓					✓	✓		✓		
(Smith, Dimiti et al. 2014) [42]																
✓		✓	✓								✓	✓		✓		
(de Vries, Hamad et al. 2021) [31]																
✓		✓	✓								✓					
(Draiko, McKague et al. 2021) [32]																
✓										✓	✓	✓				
(Komakech, Lubogo et al. 2020) [35]																
✓			✓									✓				
**Self-care for HIV**																
(Conserve, Michel et al. 2020) [30]																
	✓	✓						✓			✓	✓	✓	✓		
(Maatouk, El Nakib et al. 2021) [38]																
✓						✓						✓	✓		✓	
(O’Laughlin, He et al. 2018) [39]																
	✓	✓	✓													
(Sibanda, d’Elbee et al. 2019) [41]																
	✓		✓									✓		✓		
(Tonen-Wolyec, Batina-Agasa et al. 2019) [44]																
✓	✓						✓				✓					
(Tonen-Wolyec, Batina-Agasa et al. 2019) [45]																
	✓	✓	✓					✓				✓		✓		
(Tonen-Wolyec, Kayembe Tshilumba et al. 2020) [47]																
✓	✓		✓								✓			✓		
(Tonen-Wolyec, Mbopi-Keou et al. 2019) [46]																
		✓									✓					
(Tonen-Wolyec, Mboumba Bouassa et al. 2020) [48]																
		✓	✓													
**Self-care for gender-based violence**																
(Tol, Leku et al. 2020) [43]																
✓							✓				✓	✓		✓		
**Self-care for contraception**																
(Ammerdorffer, Laws et al. 2021) [26]																
	✓				✓				✓							
(Bertrand, Bidashimwa et al. 2018) [28]																
	✓	✓				✓					✓	✓		✓		
**Self-care for abortion**																
(Berry-Bibee, St Jean et al. 2018) [27]																
		✓			✓											
(Gill and Tam 2021) [33]																
		✓	✓										✓			
**Self-care and health services**																
(Logie, Abela et al. 2021) [37]																
	✓										✓		✓			

### Self-care for mothers and newborns

We identified ten publications from nine independent studies on self-care interventions or behaviours in crisis-affected settings. Of the ten MNH publications, six (four full articles and two abstracts) focused on maternal health interventions and four (all full articles) on newborn care interventions or behaviours.

All six maternal health publications reported on evaluations of advance distribution of misoprostol, a uterotonic pill, to address postpartum haemorrhage when women give birth outside of health facilities. Three of the four full articles reported on interventions in Afghanistan [24, 34, 40], and one in South Sudan [42]; abstracts reported aspects of the same intervention in Haiti [29, 36]. Full articles evaluated feasibility [24, 34, 40, 42], acceptability [24, 34, 40, 42] and effectiveness [24, 34, 40, 42] of distribution of misoprostol to women for self-administration immediately after birth. Abstracts focused on feasibility [29] and acceptability [36] of a similar intervention in Haiti. In all cases, distribution of tablets and counselling on their use took place during community health worker home visits [24, 34, 40, 42] or at antenatal care consultations at health facilities [29, 36, 42] Studies in Afghanistan and South Sudan found that more than 90% of women who received misoprostol and gave birth at home used the drug, and nearly all reported use of the correct number of tablets, correct timing and recall of messages [24, 34, 42].

All of the newborn health publications focused on essential newborn care, including thermal care, clean cord care and breastfeeding practices. Study locations included North-East Nigeria (Borno State) [25], occupied Palestinian Territories (Gaza) [31], South Sudan [32], and refugee settlements in Uganda [35]. Studies in Nigeria and Uganda were exploratory assessments of the acceptability and implementation of global best practices for newborn care, independent of an intervention. In the occupied Palestinian Territories, Devries et al. examined breastfeeding practices among households receiving postnatal home visits. In South Sudan, Draiko et al. evaluated the effectiveness of an intervention providing pregnant women with chlorhexidine gel for newborn cord care at home.

Studies identified several factors influencing self-care practices and outcomes, all of which reinforce that self-care interventions are part of a broader healthcare ecosystem. In all maternal health studies reporting on self-administration of misoprostol for postpartum haemorrhage prevention, drug distribution was accompanied by counselling from a trained health worker and detailed instructions on how and when to administer it. Counselling also emphasized the importance of having skilled health personnel attending childbirth and that misoprostol should only be self-administered if women cannot reach a facility or there is no skilled health provider present at a home birth. In Afghanistan and South Sudan studies, misoprostol pills were packed in boxes designed by the Ministry of Health with clear messaging and pictorial instructions for use. In Afghanistan, community health councils were also engaged to raise awareness of the intervention. Family members were engaged in the counselling process; participants noted this helped reinforce messages and ensure at least one support person was involved in birth preparedness in each household. Similarly, in Haiti, the role of health workers and community members in creating an enabling environment for misoprostol self-administration was noted. Lathrop et al. also noted positive health system benefits of the intervention, including strengthening pregnant women’s trust in community health workers and fostering closer working relationships between health centres, community health workers, traditional birth attendants and community leaders.

Authors of the study evaluating an intervention promoting the use of chlorhexidine gel for newborn cord care in South Sudan highlighted similar intervention characteristics and health system interactions as contributing to an enabling environment for intervention success – namely the fact that a majority of women attended at least two antenatal care visits with skilled health personnel, the packaging of the gel as part of a safe delivery kit distributed during pregnancy, and the engagement of Ministry of Health and community leaders in intervention design and roll-out. In the study evaluating newborn care practices among women receiving postnatal home visits in Gaza, the home visitors were identified as strong agents of change in challenging both community and healthcare provider beliefs about the benefits of artificial milk and promoting the adoption of exclusive breastfeeding, clean cord care, and hygiene practices. Studies examining newborn care in the absence of an intervention also highlighted the influence of family members (e.g., grandmothers, mothers-in-law) in thermal care, cord care and breastfeeding practices. In the Borno State of Nigeria, acceptance of recommended thermal care practices was hindered by traditional beliefs in the importance of early and frequent bathing (that body fluids cause odour later in life).

### Self-care for HIV

Out of nine HIV-related studies, eight examined HIV self-testing (HIVST), and one investigated home-based HIV testing. The Democratic Republic of Congo (DRC) was the location of five studies, which were authored by the same first author and other co-authors [44–48]. The other studies originated from Haiti [30], Lebanon [38], rural Zimbabwe [41], Uganda (in a refugee settlement) [39].

In the DRC, Tonen-Wolyec et al. compared the ability of female sex workers to read and interpret the results of HIVST compared to non-sex workers. They found that the higher educational level was a predictor of correct interpretation—sex work status was not an associated factor [44]. In a study that compared unassisted (home-based) HIVST with directly-assisted HIVST (demonstration followed by supervised self-test) among adult participants, it was found that both approaches had high rates of successfully performing the HIV self-test and correctly interpreting its results [47]. The three other studies by Tonen-Wolyec et al. focused on adolescents and university students. They found that peer-to-peer facilitated HIVST was highly acceptable and effective in yielding correct results [45], with acceptability higher among younger than older students [46] and preference for home-based vs. facility-based HIVST [48]. However, most participants found it essential to access post-test and preferably face-to-face counselling.

A study from Haiti by Conserve et al. explored the perspectives of women living with HIV, their partners, and representatives from the Ministry of Health and relevant NGOs to inform future HIVST programs in which women living with HIV would deliver HIVST kits to their partners [30]. Results highlighted the advantage of having more people learn about their HIV status and starting treatment and the risks of violence against the women distributing such kits. Equally important was the need to couple HIVST distribution with public information, education, and communication through media and social marketing, and rely on community health workers instead of women to facilitate the use of HIVST and ensure linkage to care.

In Lebanon, Maatouk et al. evaluated the implementation of HIVST kits distributed by NGO partners to men who have sex with men [38]. The program did not report any breach of confidentiality, forceful testing, or involuntary notification. However, it was found that many recipients distributed the tests to their peers or sex partners without further information and contact, which compromised programmatic follow-up.

Sibanda et al. demonstrated the cost-effectiveness of a community-led HIVST program in rural Zimbabwe, where HIVST distributors were trained over 3 days according to the Ministry of Health guidelines and given HIVST kits for distribution [41]. Training included information on HIV testing, supporting others to use HIVST kits, promoting and supporting linkage to appropriate post-test services, and providing information on the effectiveness of antiretroviral therapy for HIV prevention.

O’Laughlin et al. tested a home-based HIV program in a Ugandan refugee settlement, where research assistants with prior HIV counselling and testing mediated the process. The approach received broad acceptance, as reflected in the 75% of eligible adults who undertook HIV testing and received their results [39].

### Self-care for gender-based violence, contraception, and abortion

We identified one study concerned with self-care in the context of GBV [43]. Tol et al. conducted a cluster randomised trial to assess the effectiveness of implementing a facilitator-guided, group-based self-help intervention to reduce psychological distress among Sudanese refugees living in rural settlements in northern Uganda. This positive coping programming was delivered to groups in workshops using audio recordings and an illustrated self-help book. Improvements were noted in distress levels 3 months after the intervention among refugees with different trauma exposure, time in settlements, and initial psychological distress.

There was a focus on contraception in conflict settings in Lebanon and the DRC in two studies [26, 28]. Bertrand et al. [28] examined the acceptability and feasibility of s*elf-injection* of *subcutaneous DMPA-SC* in the DRC. This prospective cohort study found that 80% of the 640 participants reported feeling ready and confident to self-inject after training by student nurses and doctors and at 3 and 6 months, 97% described it as easy. Women were able to practice injection on a thick piece of foam mimicking skin at a community outreach event beside a health centre and community workers promoted the campaign days to identify women and follow up DMPA-SC acceptors to receive a second dose. The study by Ammerdorf et al. [26] involved a review of the Lebanese National Drug Index, National Drugs Database and Ministry of Public Health website and found that there was no official procedure to change prescription-only medicines such as oral contraception pills, DMPA-SC and abortion medications to over-the-counter (OTC). Ulipristal acetate was available OTC but the Ministry of Health did not currently provide it; rather this medication can be purchased at pharmacies.

Two studies focused on self-managed abortion [27, 33]. A study from Haiti examined the availability of misoprostol for self-managed medical abortion [27]. The interviewed women noted that their male partner was the primary person who instructed them how to take the pills and who purchased the medicine from street vendors. Women also reported using misoprostol either alone or as a part of the medication/herb regimen for their self-managed abortion (85.1%, *n* = 63). A mixed-methods study involving Venezuelan women found that most supported a digital tool to facilitate self-managed abortions safely and contraception access [33].

One paper included in the review provided insight into the knowledge and views of health professionals regarding self-care in SRH [3]. The data of survey respondents from crisis settings in Afghanistan, Lebanon, Somalia, Sudan, Syria, and Yemen were included. These respondents acknowledged the benefits of self-care and the need for linked care via hotlines, apps, home visits, and transport to facilities in case of complications. The need for provider training on interventions was identified, particularly in relation to dealing with side-effects and complications. Issues with stigma in pharmacies were noted in Syria.

## Discussion

The aim of this scoping review was to synthesize evidence regarding SRH self-care interventions and behaviours in humanitarian and fragile contexts. Across the 25 included publications, we found examples of self-care from more than ten different countries affected by conflict, forced displacement, extreme weather events, or complex emergencies. Collectively, the findings demonstrate that there is potential for well-supported self-care interventions to expand access to quality SRH for crisis-affected populations. However, descriptions of interventions, study settings and factors influencing implementation yield limited insights on how practical considerations for SRH self-care interventions may differ in stable and fragile or crisis-affected contexts.

Included studies evaluated mental health self-help techniques for GBV survivors, HIV self-testing, self-administration of injectable contraception, self-managed abortion, self-administration of misoprostol for addressing postpartum haemorrhage, and home-based essential newborn care. All were studied in isolation, and not as part of multifaceted strategies or programs to maintain or expand access to SRH services. To some extent, this reflects broader trends in research on SRH in humanitarian settings. Recent reviews of SRH service delivery in humanitarian settings note a lack of research on effective delivery strategies for SRH interventions in conflict-affected settings, especially in areas where health infrastructure has been damaged or services limited [49, 50]. Authors examining evidence of effectiveness and utilization of SRH services also found few studies describing how interventions are coordinated or evaluating interventions as part of a coordinated package of SRH services [51, 52].

A recent WHO-led initiative to identify sexual, reproductive, maternal, newborn, child, and adolescent health research priorities for humanitarian settings called for investments in implementation research that goes beyond evaluating intervention effectiveness to explore what works where, why and how. Specific priorities included testing MNH and family planning service delivery strategies (including task shifting and self-care) in different settings and phases of emergency [53]. Across the 25 self-care publications we reviewed, few interventions were studied in multiple contexts and even fewer with similar study designs and outcomes of interest enabling comparison of conditions that facilitate or hinder self-care in different settings.

We set out to identify and synthesize factors that influence the implementation and uptake of self-care interventions and the experience of individuals involved in self-care practices or support for SRH self-care in crisis-affected contexts. As Table 2 shows, most considerations highlighted were related to health worker and community member awareness, engagement and motivation in supporting self-care interventions. Previous research has shown that training and engaging community-based personnel can facilitate SRH and other primary care services in humanitarian settings. Still, there is a need for more evidence and guidance on how best to implement such a strategy and for which interventions this is feasible [50, 52, 54].

Advancing integration of self-care into health systems is complex and requires continuous efforts to meet multiple divergent demands and nuances [55]. The enabling environment and ecosystem characteristics described in the 25 self-care publications reviewed are similar to those highlighted in global surveys and studies from non-humanitarian settings [5, 56, 57]. However, the lack of discussion on commodity security and financing considerations was surprising. Other reviews and case studies have highlighted safety and security constraints, movement restrictions, forced displacement, supply chain disruptions, and shortages of skilled health professionals as barriers to SRH intervention delivery in conflict-affected settings [49, 50, 58]. None of the studies we reviewed mentioned barriers to self-care or implementation considerations that are specific to humanitarian contexts or ways in which strategies for introduction, promotion, monitoring, support or sustainability of self-care were tailored to account for health system disruptions or humanitarian access constraints. This may be because although studies took place in geographies with appeals for humanitarian assistance at the time of the study, very few described the setting as crisis-affected and or set out to examine feasibility, acceptability or effectiveness of support for self-care as a strategy for addressing humanitarian health needs.

To our knowledge, this is the first review to focus on SRH self-care in humanitarian and fragile settings. However, this scoping review is limited by its descriptive approach and can only provide an overview of studies reported in available peer-reviewed papers and abstracts. This review did not include grey literature that may have revealed insights from the field but these may also lack methodological rigour. While the authors endeavoured to search all known relevant databases, selection bias is possible if data was missed, affecting the descriptive account of available information.

Although we identified self-care examples contributing to each of the life-saving objectives of the MISP, many other SRH self-care interventions could be supported alongside tested interventions [16]. Several studies excluded from the review were identified in crisis-affected countries but not years or parts of the country with a humanitarian funding appeal or response plan [59–63]. These studies can also provide valuable country-specific insights, particularly in crisis-affected countries such as Ethiopia, Nigeria, and Uganda which have recently launched national guidelines on self-care for SRH [64].

### Research implications

Our findings offer a limited understanding of the design, implementation, and outcomes of SRH self-care interventions in humanitarian and fragile settings. Further research is needed to examine and compare different models for implementing SRH self-care while accounting for specific population needs and health system constraints. Equally important is research into other SRH self-care components that have not yet been reported in the extant literature. The clinical objectives of the MISP and self-care interventions that align with the MISP objectives could guide such research [16]. For instance, research on HIV-related interventions could include HIV post-exposure prophylaxis for sexual violence survivors or continuation of antiretroviral treatment for people already enrolled in such treatment, including pregnant and postpartum women. Research on self-care interventions for sexual violence survivors could examine community-facilitated and self-initiated administration of emergency contraception, HIV post-exposure prophylaxis, and STI presumptive treatment. Research on such models could also benefit women self-managing medical abortion in the first trimester and the initiation of post-abortion contraception.

## Conclusions

There is a lack of evidence on the design, implementation, and outcomes of SRH self-care interventions in humanitarian and fragile settings. Articles reporting on self-care interventions in countries affected by fragility or humanitarian crises provide little information on experiences of consumers, factors affecting the implementation and uptake of self-care interventions, or barriers to the use of self-care interventions. The time to invest in self-care implementation research is now, ensuring that implementation support is extended to crisis-affected areas, and countries developing emergency preparedness and response or self-care guidelines have lessons to draw on. Whether expanding support for established self-care interventions to crisis-affected populations or introducing new models of care, better descriptions of how promotion and support of self-care are tailored to context-specific population interests and health system considerations are needed to inform programmatic guidance.

## Supplementary Information

**Additional file 1.** Humanitarian Response Plans since 2002 in fragile and conflict-affected countries according to crisis and date.

**Additional file 2.** Database Searches.

**Additional file 3.** Preferred Reporting Items for Systematic reviews and Meta-Analyses extension for Scoping Reviews (PRISMA-ScR) Checklist.

## Data Availability

All data generated and analysed during the current study are available from the corresponding author on reasonable request.

## Abbreviations

DMPA-SC: Depot medroxyprogesterone acetate subcutaneous injectable contraception
DRC: The Democratic Republic of the Congo
GBV: Gender-based violence
HIV: Human immunodeficiency virus
HIVST: HIV self-testing
HRP: Humanitarian Response Plan
HPV: Human papilloma virus
MISP: Minimum Initial Services Package for Reproductive Health in Crises
MNH: Maternal and newborn health
OTC: Over-the-counter
SRH: Sexual and reproductive health
STI: Sexually transmitted infection
WHO: World Health Organization
