# Supplementary material for: Self-care interventions for sexual and reproductive health in humanitarian and fragile settings: a scoping review
Source: BMC Health Serv Res. 2022 Jun 7;22:757. doi: 10.1186/s12913-022-07916-4 (PMC9172979; doi:10.1186/s12913-022-07916-4)
Supplement: Supplementary file 1 — Additional file 1. Humanitarian Response Plans since 2002 in fragile and conflict-affected countries according to crisis and date. [file 12913_2022_7916_MOESM1_ESM.docx]

**Additional file 1: Humanitarian Response Plans since 2002 in fragile and conflict-affected countries according to crisis and date**

| **CRISIS** | **LOCATION/ REGION** | **DATES OF CRISIS** | **REFERENCES to HRPs** |
| --- | --- | --- | --- |
| **HIGH-INTENSITY CONFLICT** | | |  |
| **AFGHANISTAN** | | |  |
| Civil war,  drought |  | Since 1992 | <https://www.humanitarianresponse.info/en/operations/afghanistan/document/afghanistan-humanitarian-response-plan-2018-2021-2021-revision>  <https://www.humanitarianresponse.info/en/operations/afghanistan/document/afghanistan-2017-humanitarian-response-plan-mid-year-review-january>  <https://reliefweb.int/report/afghanistan/afghanistan-2016-humanitarian-response-plan-january-december-2016>  <https://www.humanitarianresponse.info/en/programme-cycle/space/document/afghanistan-2015-strategic-response-plan>  <https://www.humanitarianresponse.info/en/programme-cycle/space/document/chap-2014-strategic-response-plan> Afghanistan <https://reliefweb.int/report/afghanistan/afghanistan-common-humanitarian-action-plan-2013>  <https://reliefweb.int/report/afghanistan/afghanistan-2009-humanitarian-action-plan>  <https://reliefweb.int/report/afghanistan/strategy-assistance-community-response-drought-afghanistan-1-jun-2000-31-may-2001> |
| **LIBYA** |  |  |  |
| Civil war |  | Since 2011 |  |
|  |  | HRP 2016-2021 | <https://www.humanitarianresponse.info/en/programme-cycle/space/document/2016-libya-humanitarian-response-plan> first |
| **SOMALIA** |  |  |  |
| Somali civil war |  | 1988-1995 | United Nations Operation in Somalia I & 11 1992-1995 |
| Somalia war | Somaliland and Puntland | 2006–2009 |  |
| Civil war | Southern & Central Somalia | 2009-ongoing, HRPs from 2015 | <https://reliefweb.int/report/somalia/somalia-humanitarian-response-plan-2021-february-2021>  <https://reliefweb.int/report/somalia/somalia-humanitarian-response-plan-2020-hrp-revision-covid-19-july-2020>  <https://reliefweb.int/report/somalia/2019-somalia-humanitarian-response-plan-summary-enso>  <https://reliefweb.int/report/somalia/somalia-humanitarian-response-plan-revised-july-december-2018>  https://reliefweb.int/report/somalia/somalia-humanitarian-response-plan-may-2017-revision  [https://www.humanitarianresponse.info/en/operations/somalia/document/2017-somalia-humanitarian-response-plan first HRP 2017](https://www.humanitarianresponse.info/en/operations/somalia/document/2017-somalia-humanitarian-response-plan%20first%20HRP%202017)  <https://reliefweb.int/report/somalia/somalia-humanitarian-response-plan-january-december-2016>  <https://reliefweb.int/report/somalia/somalia-humanitarian-response-plan-2015> |
| **SYRIA** |  |  |  |
| Civil war |  | 2011- ongoing, HRPs since 2012 | <https://www.humanitarianresponse.info/en/operations/whole-of-syria/document/overview-2016-syria-humanitarian-response-plan-2016-2017-regional>  <https://reliefweb.int/report/lebanon/syria-regional-response-plan-january-december-2013>  <https://reliefweb.int/report/jordan/syria-regional-response-plan-march-2012> |
| **MEDIUM-INTENSITY CONFLICT** |  |  |  |
| **BURKINA FASO** |  |  |  |
| Sahel Crisis: |  | 2011-2017 | <https://www.humanitarianresponse.info/en/operations/west-and-central-africa/document/wash-nut-strategy> |
| Terrorism displacing more than 100,000 people | [Hauts-Bassins](https://en.wikipedia.org/wiki/Hauts-Bassins_Region), [Boucle du Mouhoun](https://en.wikipedia.org/wiki/Boucle_du_Mouhoun_Region), [Nord](https://en.wikipedia.org/wiki/Nord_Region_(Burkina_Faso)), [Sahel](https://en.wikipedia.org/wiki/Sahel_Region), and [Est](https://en.wikipedia.org/wiki/Est_Region_(Burkina_Faso)) regions | 2015- 2021 HRPs |  |
|  | Boucle du Mouhoun: [Kossi](https://en.wikipedia.org/wiki/Kossi_Province), [Sourou](https://en.wikipedia.org/wiki/Sourou_Province), Centre-Est: [Koulpélogo](https://en.wikipedia.org/wiki/Koulp%C3%A9logo_Province)  Est: [Gnagna](https://en.wikipedia.org/wiki/Gnagna_Province), [Gourma](https://en.wikipedia.org/wiki/Gourma_Province), [Komandjari](https://en.wikipedia.org/wiki/Komondjari_Province), [Kompienga](https://en.wikipedia.org/wiki/Kompienga_Province), [Tapoa](https://en.wikipedia.org/wiki/Tapoa_Province)  Hauts-Bassins: [Kénédougou](https://en.wikipedia.org/wiki/K%C3%A9n%C3%A9dougou_Province)  Nord: [Lorum](https://en.wikipedia.org/wiki/Loroum_Province), Sahel: [Oudalan](https://en.wikipedia.org/wiki/Oudalan_Province), [Séno](https://en.wikipedia.org/wiki/S%C3%A9no_Province), [Soum](https://en.wikipedia.org/wiki/Soum_Province), [Yagha](https://en.wikipedia.org/wiki/Yagha_Province) | State of Emergency 2018 |  |
|  |  | 2015 -21 HRPs | <https://www.humanitarianresponse.info/en/programme-cycle/space/document/burkina-faso-srp-2015-plan-de-r%C3%A9ponse-strat%C3%A9gique-2015> |
| **CAMEROON** |  |  |  |
|  | North-West, South-West, Littoral, West and Centre regions | 2020-21 | <https://www.humanitarianresponse.info/en/operations/cameroon/document/cameroon-humanitarian-response-plan-2021>  <https://www.humanitarianresponse.info/en/op%C3%A9rations/cameroon/document/cameroon-humanitarian-response-plan-2020-revised> |
|  | Adamaoua, East, North and Far-North | 2018 | <https://www.humanitarianresponse.info/en/operations/cameroon/document/cameroon-humanitarian-response-plan%C2%A02018%C2%A0summary>  <https://www.humanitarianresponse.info/en/operations/west-and-central-africa/document/plan-de-r%C3%A9ponse-humanitaire-cameroun-2017> |
|  | Adamaoua, East, North and Far-North | Dec 2012 | <https://www.humanitarianresponse.info/en/operations/chad/document/central-african-republic-regional-response-plan-january-december-2014> |
|  | Lake Chad Basin | 2017 | <https://www.humanitarianresponse.info/en/operations/nigeria/document/lake-chad-basin-emergency-humanitarian-needs-and-requirement-overview> |
| **CENTRAL AFRICAN REPUBLIC** |  |  |  |
| Civil war coup in December. 2012 | Boda-Mbaiki-Batalimo Bangui-Damara | Dec 2012 | <https://www.humanitarianresponse.info/en/operations/chad/document/central-african-republic-regional-response-plan-january-december-2014>  <https://www.humanitarianresponse.info/en/operations/central-african-republic/document/central-african-republic-strategic-response-plan-2014>  <https://www.humanitarianresponse.info/en/operations/central-african-republic/document/100-day-plan-priority-humanitarian-action-rca> |
|  |  | Since 2017 | <https://reliefweb.int/report/central-african-republic/central-african-republic-humanitarian-response-plan-2021>  <https://www.humanitarianresponse.info/sites/www.humanitarianresponse.info/files/2019/02/2018_hrp_CAR_english_final_0.pdf>  <https://www.humanitarianresponse.info/en/operations/central-african-republic/document/rca-ocha-executive-summary-hrp-2017>  <https://www.humanitarianresponse.info/en/operations/central-african-republic/document/rca-iasc-plan-de-r%C3%A9ponse-humanitaire-nov-2016> |
| **CHAD** |  |  |  |
| Conflict related to Boko Haram Islamist insurgency |  | Since 2010  But HRPs since 2014 | <https://www.humanitarianresponse.info/en/operations/cameroon/document/nigeria-regional-refugee-response-plan-2019-2020>  <https://www.humanitarianresponse.info/en/operations/chad/infographic/chad-response-plan-2018-revised-onee-pager> |
|  |  |  | <https://www.humanitarianresponse.info/en/operations/chad/document/chad-press-release-launching-humanitarian-response-plan-2018-9-february>  <https://www.humanitarianresponse.info/en/operations/cameroon/document/nigeria-regional-refugee-response-plan-glance-january-december-2017>  [https://www.humanitarianresponse.info/en/operations/nigeria/document/lake-chad-basin-emergency-humanitarian-needs-and-requirement-overview 2017](https://www.humanitarianresponse.info/en/operations/nigeria/document/lake-chad-basin-emergency-humanitarian-needs-and-requirement-overview%202017)  <https://www.humanitarianresponse.info/en/operations/chad/infographic/chad-90-day-emergency-response-plan-crisis-lac-region-1st-july-%E2%80%93-30>  <https://www.humanitarianresponse.info/en/operations/chad/document/tchad-hrp-2016-plan-de-r%C3%A9ponse-humanitaire-2016-chad>  <https://www.humanitarianresponse.info/en/operations/chad/document/tchad-hrp-2015-plan-de-r%C3%A9ponse-humanitaire-2015>  <https://www.humanitarianresponse.info/en/programme-cycle/space/document/revision-strategic-response-plan-republic-chad-2014-2016-august-2014> |
| **CONGO, DEM. REP** |  |  |  |
| A complex emergency has persisted in DRC for more than 20 years. <https://www.acaps.org/country/drc/crisis/complex-crisis>   DR Congo: Ebola Outbreak - Feb 2021   [DR Congo: Volcano Nyiragongo - May 2021](https://www.humanitarianresponse.info/en/operations/democratic-republic-congo/documents/table/disasters/263627)   [DR Congo: Ebola Outbreak - Aug 2018](https://www.humanitarianresponse.info/en/operations/democratic-republic-congo/documents/table/disasters/168601)   [DR Congo: Ebola Outbreak - Aug 2014 (](https://www.humanitarianresponse.info/en/operations/democratic-republic-congo/documents/table/disasters/33174)   DR Congo: Ebola Outbreak - May 2018   [West Africa: Ebola Outbreak 2014 - 2015](https://www.humanitarianresponse.info/en/operations/democratic-republic-congo/documents/table/disasters/33204)   [DR Congo: Floods - Apr 2020](https://www.humanitarianresponse.info/en/operations/democratic-republic-congo/documents/table/disasters/238229)   [DR Congo: Ebola Outbreak - Jun 2020](https://www.humanitarianresponse.info/en/operations/democratic-republic-congo/documents/table/disasters/246040)   [DR Congo: Floods - Oct 2019](https://www.humanitarianresponse.info/en/operations/democratic-republic-congo/documents/table/disasters/203508)   [DR Congo: Floods - Nov 2016 (2)](https://www.humanitarianresponse.info/en/operations/democratic-republic-congo/documents/table/disasters/136511) |  | 2007-2021 HRPs | <https://www.humanitarianresponse.info/en/operations/democratic-republic-congo/document/rd-congo-plan-de-r%C3%A9ponse-humanitaire-2021>  <https://www.humanitarianresponse.info/en/op%C3%A9rations/d%C3%A9mocratic-republic-congo/infographic/drc-humanitarian-dashboard-1e-trimestre-2020>  <https://reliefweb.int/report/democratic-republic-congo/dr-congo-2017-2019-humanitarian-response-plan-summary-update-2018>  <https://reliefweb.int/report/democratic-republic-congo/dr-congo-2017-2019-humanitarian-response-plan-2018-update>  <https://www.humanitarianresponse.info/en/operations/democratic-republic-congo/document/rdc-plan-de-r%C3%A9ponse-humanitaire-2016>  <https://www.humanitarianresponse.info/en/programme-cycle/space/document/rdc-plan-de-r%C3%A9ponse-humanitaire-2015>  <https://www.humanitarianresponse.info/en/programme-cycle/space/document/plan-de-r%C3%A9ponse-strat%C3%A9gique-pour-la-r%C3%A9publique-d%C3%A9mocratique-du-congo>  <https://www.humanitarianresponse.info/en/programme-cycle/space/document/plan-daction-humanitaire-pour-la-r%C3%A9publique-d%C3%A9mocratique-du-congo-0>  <https://www.humanitarianresponse.info/en/programme-cycle/space/document/mid-year-review-humanitarian-action-plan-2012-democratic-republic>  <https://www.humanitarianresponse.info/en/programme-cycle/space/document/humanitarian-action-plan-democratic-republic-congo-2011>  <https://reliefweb.int/report/democratic-republic-congo/humanitarian-action-plan-2010-democratic-republic-congo>  <https://reliefweb.int/report/democratic-republic-congo/humanitarian-action-plan-2009-democratic-republic-congo>  <https://www.humanitarianresponse.info/en/programme-cycle/space/document/action-plan-democratic-republic-congo-2008>  <https://www.humanitarianresponse.info/en/programme-cycle/space/document/democratic-republic-congo-humanitarian-action-plan-2007>  <https://www.humanitarianresponse.info/en/programme-cycle/space/document/action-plan-democratic-republic-congo-2006> |
| **IRAQ** |  |  |  |
| Civil unrest US invasion & occupation 2003-2008  Since 2008 ISIL & instability & unrest |  | Since 2014 HRPs | <https://www.humanitarianresponse.info/en/operations/iraq/document/iraq-2021-humanitarian-response-plan-february-2021-en>  <https://www.humanitarianresponse.info/en/operations/iraq/document/iraq-2020-humanitarian-response-plan-en>  <https://www.humanitarianresponse.info/en/operations/iraq/document/iraq-2019-humanitarian-response-plan-january-december-2019>  <https://www.humanitarianresponse.info/en/operations/iraq/document/iraq-2019-humanitarian-response-plan-january-december-2019>  <https://www.humanitarianresponse.info/en/operations/iraq/document/iraq-humanitarian-response-plan-2018-advanced-executive-summary>  <https://www.humanitarianresponse.info/en/operations/iraq/document/2017-iraq-humanitarian-response-plan>  <https://www.humanitarianresponse.info/en/operations/iraq/document/2016-iraq-humanitarian-response-plan>  <https://www.humanitarianresponse.info/en/operations/iraq/document/emergency-response-preparedness-2015-final>  <https://www.humanitarianresponse.info/en/programme-cycle/space/document/iraq-strategic-response-plan-srp-2014-2015>  <https://www.humanitarianresponse.info/en/programme-cycle/space/document/iraq-strategic-response-plan-srp-revision-june-2014> |
| **MALI** |  |  |  |
| Civil unrest | January 2012 [a Tuareg rebellion](https://en.wikipedia.org/wiki/Tuareg_rebellion_(2012)) began in Northern Mali  central Mali province of [Mopti](https://en.wikipedia.org/wiki/Mopti_Region), conflict has escalated since 2015 | HRPs 2016-2021 | <https://www.humanitarianresponse.info/en/operations/mali/plan-de-r%C3%A9ponse-humanitaire>  <https://www.humanitarianresponse.info/en/operations/mali/document/mali-plan-de-r%C3%A9ponse-humanitaire-janvier-d%C3%A9cembre-2021>  <https://reliefweb.int/report/mali/mali-crisis-response-plan-2020>  <https://www.humanitarianresponse.info/en/operations/mali/document/mali-plan-de-r%C3%A9ponse-humanitaire-janvier-decembre-2019>  <https://www.globalprotectioncluster.org/_assets/files/hrp-2018_mali_en_20180222_vf_0.pdf>  <https://www.humanitarianresponse.info/en/operations/mali/document/plan-de-reponse-humanitaire-2016-pour-le-mali> |
| **MOZAMBIQUE** |  |  |  |
| a low-intensity insurgency by RENAMO |  | 2013 to 2019 | None identified |
| Ongoing insurgency by Islamist groups | north East Cabo Delgado province | Since 2015 | None identified |
| Natural disasters |  | 2021 HRP  2008-2010 HRPs | <https://www.humanitarianresponse.info/en/operations/mozambique/document/mozambique-humanitarian-response-plan-2021-abridged-version>  [https://www.humanitarianresponse.info/en/operations/mozambique/document/mozambique-hct-inter-agency-contingency-plan-2008-2009 2008-2009](https://www.humanitarianresponse.info/en/operations/mozambique/document/mozambique-hct-inter-agency-contingency-plan-2008-2009%202008-2009)  <https://www.humanitarianresponse.info/en/operations/mozambique/document/mozambique-hct-inter-agency-contingency-plan-2009-2010> |
| Cyclones Idai and Kenneth |  | 2018-2019 | <https://www.humanitarianresponse.info/en/operations/mozambique/document/2018-2019-mozambique-humanitarian-response-plan-revised-following> |
| Flood |  | 2000 Feb-March | None identified |
| Drought | Southern and Central region Tete, Sofala, Gaza, Inhambane, Manica, Zambezia and Maputo | 2016 | <https://www.humanitarianresponse.info/en/operations/mozambique/document/mozambique-strategic-drought-response-plan-2016>  <https://www.humanitarianresponse.info/en/operations/mozambique/document/mozambique-strategic-response-plan-2016> |
| **MYANMAR** |  |  |  |
| Rohingya refugees & IPD | Rakhine, Kachin and Shan states | 2012,2016, 2017 | <https://www.humanitarianresponse.info/en/operations/bangladesh/document/2020-joint-response-plan-rohingya-humanitarian-crisis-january-0>  <https://www.humanitarianresponse.info/en/operations/bangladesh/infographic/2020-joint-response-plan-rohingya-humanitarian-crisis-snapshot>  <https://www.humanitarianresponse.info/en/operations/bangladesh/document/2019-joint-response-plan-rohingya-humanitarian-crisis-january>  <https://www.humanitarianresponse.info/en/operations/bangladesh/document/humanitarian-response-plan-september-2017-february-2018-final-report>  <https://www.humanitarianresponse.info/en/operations/bangladesh/document/jrp-rohingya-humanitarian-crisis>  <https://www.humanitarianresponse.info/en/operations/bangladesh/document/bangladesh-humanitarian-response-plan-september-2017-february-2018>  <https://www.humanitarianresponse.info/en/operations/myanmar/document/myanmar-humanitarian-response-plan-2017> |
| Flooding |  | 2015 | <https://www.humanitarianresponse.info/en/programme-cycle/space/document/2016-myanmar-humanitarian-response-plan>  <https://www.humanitarianresponse.info/en/programme-cycle/space/document/2015-myanmar-initial-flood-response-plan-august-december-2015>  <https://www.humanitarianresponse.info/en/programme-cycle/space/document/2015-myanmar-humanitarian-response-plan> |
| **NIGER** |  |  |  |
| Complex crisis | Maradi Boko Haram Islamist insurgency in the south east  Tillabery region Jihadi militant since 2019 | 2015-2021  HRP-2014-2021 | <https://www.humanitarianresponse.info/en/operations/west-and-central-africa/document/sahel-2021-sahel-overview-humanitarian-needs-and>  <https://www.humanitarianresponse.info/en/operations/niger/document/niger-plan-de-r%C3%A9ponse-humanitaire-2021>  <https://www.humanitarianresponse.info/en/op%C3%A9rations/niger/document/niger-plan-de-r%C3%A9ponse-humanitaire-2020>  <https://www.humanitarianresponse.info/en/operations/niger/document/niger-plan-de-r%C3%A9ponse-humanitaire-2019>  <https://www.humanitarianresponse.info/en/operations/niger/document/niger-cadre-strategique-operationnel-2018>  <https://www.humanitarianresponse.info/en/operations/niger/document/niger-plan-de-r%C3%A9ponse-humanitaire-2018>  <https://www.humanitarianresponse.info/en/operations/niger/document/niger-plan-de-r%C3%A9ponse-humanitaire-2017>  <https://www.humanitarianresponse.info/en/operations/niger/document/niger-plan-de-r%C3%A9ponse-humanitaire-2017-r%C3%A9vis%C3%A9>  <https://www.humanitarianresponse.info/en/operations/niger/document/niger-plan-de-r%C3%A9ponse-humanitaire-2016-jan-2016>  <https://www.humanitarianresponse.info/en/programme-cycle/space/document/niger-srp-2015-plan-de-r%C3%A9ponse-strat%C3%A9gique-2015>  <https://www.humanitarianresponse.info/en/operations/niger/document/sahel-2014-2016-regional-humanitarian-response-strategy-reviewed-en> |
| **NIGERIA** | | | |
| Conflict related to Boko Haram Islamist insurgency and 1.92 million people are displaced internally | Borno, Adamawa and Yobe (BAY) states in north-east Nigeria | Since 2010 but HRPs from 2014 | <https://www.humanitarianresponse.info/en/document/nigeria-2021-humanitarian-response-plan>  <https://www.humanitarianresponse.info/en/document/nigeria-2021-humanitarian-response-plan-summary>  <https://www.humanitarianresponse.info/en/operations/nigeria/document/nigeria-2020-humanitarian-response-plan> |
| Flooding | Niger, Kogi, Anambra and Delta, Rivers, Bayelsa, Taraba, Kebbi, Adamawa | Sept-Nov 2018 | <https://www.humanitarianresponse.info/en/disaster/fl-2018-000120-nga>  <https://reliefweb.int/report/nigeria/nigeria-flood-response-emergency-plan-action-final-report-emergency-appeal-n-mdrng025> |
| **SOUTH SUDAN** |  |  |  |
| Conflict  Second Sudanese Civil War 1983 to 2005  South Sudanese Civil War 2013-2020 |  | HRPs since 2014 | <https://www.humanitarianresponse.info/en/document/south-sudan-2021-humanitarian-response-plan>  <https://www.humanitarianresponse.info/en/operations/south-sudan/document/south-sudan-2020-humanitarian-response-plan-12-dec-2019>  <https://www.humanitarianresponse.info/en/operations/south-sudan/document/south-sudan-2019-humanitarian-response-plan-13-december-2018>  <https://reliefweb.int/report/south-sudan/south-sudan-2018-humanitarian-response-plan>  <https://www.humanitarianresponse.info/en/operations/south-sudan/document/south-sudan-humanitarian-response-plan-2017>  <https://www.humanitarianresponse.info/en/operations/south-sudan/document/south-sudan-humanitarian-response-plan-2016>  <https://www.humanitarianresponse.info/en/operations/south-sudan/document/south-sudan-humanitarian-response-plan-2015>  <https://www.humanitarianresponse.info/en/operations/sudan/document/sudan-strategic-response-plan-2014> |
| **YEMEN, REP** |  |  |  |
| Revolution 2011  Civil war  2014-21  Famine since 2016 |  | 2010-21 HRPs | <https://reliefweb.int/report/yemen/yemen-humanitarian-response-plan-2021-march-2021-enar>  <https://reliefweb.int/report/yemen/yemen-humanitarian-response-plan-extension-june-december-2020-enar>  <https://www.humanitarianresponse.info/en/operations/yemen/document/yemen-humanitarian-response-plan-2019-funding-status-31-december-2019-enar>  <https://www.humanitarianresponse.info/en/operations/yemen/document/yemen-humanitarian-response-plan-january-december-2018>  https://www.humanitarianresponse.info/en/operations/yemen/document/yemen2017-hrpfinal  https://www.humanitarianresponse.info/en/programme-cycle/space/document/2016-yemen-humanitarian-response-plan  <https://www.humanitarianresponse.info/en/programme-cycle/space/document/2015-yemen-humanitarian-response-plan-revision>  <https://www.humanitarianresponse.info/en/operations/yemen/document/yemen-humanitarian-response-plan-2014-2015-revision>  <https://www.humanitarianresponse.info/en/operations/yemen/document/yemen-humanitarian-response-plan-2013-0>  <https://www.humanitarianresponse.info/en/programme-cycle/space/document/humanitarian-response-plan-yemen-2012>  <https://www.humanitarianresponse.info/en/programme-cycle/space/document/humanitarian-response-plan-yemen-2011>  <https://www.humanitarianresponse.info/en/programme-cycle/space/document/humanitarian-response-plan-yemen-2010> |
| **HIGH-INSTITUTIONAL AND SOCIAL FRAGILITY** | | | |
| **NON-SMALL STATES** | | | |
| **BURUNDI** |  |  |  |
| Burundi has been in crisis since April 2015, <https://www.acaps.org/country/burundi/crisis/complex-crisis-> |  | 2019-21 HRP | <https://reliefweb.int/report/burundi/burundi-humanitarian-response-plan-2021-march-2021>  <https://reliefweb.int/report/burundi/burundi-humanitarian-response-plan-2020-funding-overview-11-december-2020>  <https://www.humanitarianresponse.info/en/operations/burundi/document/plan-de-r%C3%A9ponse-humanitaire-2019>  <http://www.fao.org/emergencies/appeals/detail/en/c/1099794/>  <https://www.humanitarianresponse.info/en/operations/burundi/document/m%C3%A9canismes-de-coordination-burundi> 2013 |
| **CONGO, REP.** |  |  |  |
| Central African Republic conflict | Refugees in the north- Likouala | Since Dec 2012 only 1 HRP 2014 | <https://www.humanitarianresponse.info/en/operations/chad/document/central-african-republic-regional-response-plan-january-december-2014> |
| Flooding | les départements de la Likouala, de la Cuvette, de la Sangha et des Plateaux. | Oct 2019 | <https://www.humanitarianresponse.info/en/operations/congo/document/plan-de-r%C3%A9ponse-aux-inondations-en-republique-du-congo> |
| **ERITREA** |  |  |  |
| Sensitive to weather changes |  |  | None identified <https://reliefweb.int/report/eritrea/humanitarian-action-children-2021-eritrea> |
| **GAMBIA, THE** |  |  |  |
| Drought spells, flooding, windstorms, pest infestation and disease outbreaks has resulted in increased food insecurity |  |  | <https://www.humanitarianresponse.info/ru/operations/gambia/document/gambia-humanitarian-response-plan-2016>  <https://www.humanitarianresponse.info/en/operations/gambia/document/gambia-response-and-recovery-plan-2013> |
| **GUINEA-BISSAU** |  |  |  |
| Natural disaster vulnerability  [Guinea-Bissau Civil War](https://en.wikipedia.org/wiki/Guinea-Bissau_Civil_War) |  | 1998-1999 | None identified |
| Ebola |  | 2014 | <https://www.humanitarianresponse.info/en/operations/west-and-central-africa/document/sub-regional-response-ebola-outbreak-affected-countries> |
| **HAITI** |  |  |  |
| Natural disasters and extreme weather events |  [Hurricane Matthew - Sep 2016](https://www.humanitarianresponse.info/en/operations/haiti/documents/table/disasters/132889)   [Haiti: Cholera Outbreak - 2010-2017 (](https://www.humanitarianresponse.info/en/operations/haiti/documents/table/disasters/59564)   [Haiti: Earthquake - Oct 2018](https://www.humanitarianresponse.info/en/operations/haiti/documents/table/disasters/171311)   [Haiti: Earthquakes - Jan 2010 (](https://www.humanitarianresponse.info/en/operations/haiti/documents/table/disasters/132321)   [Caribbean: Drought - 2015-2017](https://www.humanitarianresponse.info/en/operations/haiti/documents/table/disasters/109064)   Haiti: Floods - Apr 2021 (2)   [Hurricane Dorian - Aug 2019](https://www.humanitarianresponse.info/en/operations/haiti/documents/table/disasters/187047)   [Tropical Storm Laura - Aug 2020r](https://www.humanitarianresponse.info/en/operations/haiti/documents/table/disasters/249488)   [Central America and the Caribbean: Drought - Jul 2018 (1)](https://www.humanitarianresponse.info/en/operations/haiti/documents/table/disasters/168007)   [Hurricane Irma - Sep 2017 (1)](https://www.humanitarianresponse.info/en/operations/haiti/documents/table/disasters/151667) | 2016-21 HRP  2013 HRPs | <https://www.humanitarianresponse.info/en/operations/haiti/document/ha%C3%AFti-plan-de-r%C3%A9ponse-humanitaire-hrp-2021-2022>  <https://reliefweb.int/report/haiti/haiti-humanitarian-response-plan-january-2019-december-2020-jan-2019-summary>  <https://www.humanitarianresponse.info/ru/operations/haiti/document/haiti-2018-revised-humanitarian-response-plan-january-december-2018>  <https://reliefweb.int/report/haiti/haiti-humanitarian-response-plan-january-2017-december-2018>  <https://www.humanitarianresponse.info/en/operations/haiti/document/haiti-humanitarian-response-plan-2016>  <https://www.humanitarianresponse.info/en/operations/haiti/document/haiti-humanitarian-action-plan-2013> |
| **KOSOVO** |  |  |  |
| Kosovo conflict |  | 1998–99 | None identified |
| **LAO PDR** |  |  |  |
| Flooding | Sanamxay District | Aug-Dec 2018 | <https://reliefweb.int/report/lao-peoples-democratic-republic/lao-pdr-disaster-response-plan-august-2018-december-2018> |
| 2016 classified as authoritarian regime Hmong refugees |  |  |  |
| **LEBANON** |  |  |  |
| Lebanese Civil War  [Syrian occupation of Lebanon](https://en.wikipedia.org/wiki/Syrian_occupation_of_Lebanon)  [Syrian Civil War spillover in Lebanon](https://en.wikipedia.org/wiki/Syrian_Civil_War_spillover_in_Lebanon),  [Lebanese liquidity crisis](https://en.wikipedia.org/wiki/Lebanese_liquidity_crisis) |  | 1975 –1990  1975-2005  2011-2017  2019 |  |
|  |  | HRPs & CRPs since 2011 | <https://reliefweb.int/report/lebanon/lebanon-humanitarian-response-plan-2021>  <https://reliefweb.int/report/lebanon/lebanon-crisis-response-plan-2017-2021-2021-update>  <https://www.unhcr.org/lb/wp-content/uploads/sites/16/2019/04/LCRP-EN-2019.pdf>  <https://reliefweb.int/report/lebanon/lebanon-crisis-response-plan-2015-2016>  <https://reliefweb.int/report/lebanon/2014-syria-regional-response-plan-lebanon>  <https://reliefweb.int/report/lebanon/syria-regional-response-plan-january-december-2013>  <https://reliefweb.int/report/jordan/syria-regional-response-plan-march-2012>  <https://www.ilo.org/wcmsp5/groups/public/---arabstates/---ro-beirut/documents/publication/wcms_542062.pdf> |
| **LIBERIA** |  |  |  |
| [First Liberian Civil War](https://en.wikipedia.org/wiki/First_Liberian_Civil_War)  Second Liberian Civil War |  | 1989-1997  1999-2003 | None identified |
|  |  |  | <https://www.humanitarianresponse.info/en/operations/liberia/document/liberia-2013-critical-humanitarian-gaps> |
| Ebola |  | 2014 | <https://www.humanitarianresponse.info/en/operations/liberia/document/liberia-ebola-emergency-response-update-n1-september-5-2014>  https://www.humanitarianresponse.info/en/operations/west-and-central-africa/document/sub-regional-response-ebola-outbreak-affected-countries |
|  |  |  | <http://www.fao.org/emergencies/appels/appel-detail/fr/c/149390/> 2011 |
| **PAPUA NEW GUINEA** |  |  |  |
| Climate drought & highland frosts |  | 2016-2017 | <https://www.humanitarianresponse.info/en/operations/papua-new-guinea/document/dmt-el-nino-response-plan-18-apr-2016>  <https://www.humanitarianresponse.info/en/operations/papua-new-guinea/document/png-el-ni%C3%B1o-early-action-plan-31-jul-2017> |
| Earthquake |  | 2018 | <https://www.humanitarianresponse.info/en/operations/papua-new-guinea/document/png-75-earthquakedmt-response-plan-28-march-2018> |
| **SUDAN** |  |  |  |
| Second Sudanese Civil War  [Sudanese conflict in](https://en.wikipedia.org/wiki/Sudanese_conflict_in_South_Kordofan_and_Blue_Nile) | South Kordofan and Blue Nile | 1983 to 2005  2010 | None identified |
|  |  | 2014-2021 HRPs | <https://reliefweb.int/report/sudan/sudan-humanitarian-response-plan-2021-january-2021-enar>  <https://www.humanitarianresponse.info/en/operations/sudan/document/sudan-2020-humanitarian-response-plan>  <https://www.humanitarianresponse.info/en/operations/sudan/document/sudan-2019-humanitarian-response-plan>  <https://www.humanitarianresponse.info/en/operations/south-sudan/document/south-sudan-2018-humanitarian-response-plan>  <https://www.humanitarianresponse.info/en/operations/sudan/document/sudan-2017-humanitarian-response-plan>  <https://www.humanitarianresponse.info/en/operations/south-sudan/document/south-sudan-humanitarian-response-plan-2016>  <https://www.humanitarianresponse.info/en/operations/sudan/document/2015-sudan-humanitarian-response-plan>  <https://www.humanitarianresponse.info/en/operations/sudan/document/sudan-strategic-response-plan-2014> |
| **VENEZUELA, RB** |  |  |  |
| Crisis in Venezuela during the Bolivarian Revolution |  | 2019-2021 | <https://reliefweb.int/report/venezuela-bolivarian-republic/venezuela-humanitarian-response-plan-update-2021>  <https://www.humanitarianresponse.info/en/operations/venezuela/document/marco-de-trabajo-del-hrp-2021-cl%C3%BAster-de-nutrici%C3%B3n-venezuela>  <https://reliefweb.int/report/venezuela-bolivarian-republic/venezuela-humanitarian-response-plan-humanitarian-needs>  <https://www.humanitarianresponse.info/en/operations/venezuela/document/venezuela-humanitarian-response-plan-july-2019-%E2%80%93-december-2019> |
| **WEST BANK AND GAZA (TERRITORY)** |  |  |  |
|  |  | 2014-21 | <https://www.humanitarianresponse.info/ru/operations/occupied-palestinian-territory/document/2021-humanitarian-response-plan-occupied>  <https://www.humanitarianresponse.info/en/operations/occupied-palestinian-territory/document/2018-2020-opt-humanitarian-response-plan>  <https://www.humanitarianresponse.info/en/operations/occupied-palestinian-territory/document/2019-opt-humanitarian-response-plan>  <https://www.un.org/unispal/document/auto-insert-197428/> 2017  <https://www.humanitarianresponse.info/en/operations/occupied-palestinian-territory/document/2016-opt-humanitarian-response-plan>  <https://reliefweb.int/report/occupied-palestinian-territory/2015-humanitarian-response-plan-end-year-monitoring-update>  <https://www.un.org/unispal/document/auto-insert-201820/>  <https://www.humanitarianresponse.info/en/programme-cycle/space/document/strategic-response-plan-occupied-palestinian-territory-2014> |
| **ZIMBABWE** |  |  |  |
| Natural disaster /climate change food insecurity |  | 2016-2017 HRP  2020-2021 HRP | [https://www.humanitarianresponse.info/en/operations/zimbabwe/document/2021-zimbabwe-humanitarian-response-plan-draft https://reliefweb.int/report/zimbabwe/zimbabwe-humanitarian-response-plan-2020-march-2020](https://www.humanitarianresponse.info/en/operations/zimbabwe/document/2021-zimbabwe-humanitarian-response-plan-draft https://reliefweb.int/report/zimbabwe/zimbabwe-humanitarian-response-plan-2020-march-2020 2)  [2](https://www.humanitarianresponse.info/en/operations/zimbabwe/document/2021-zimbabwe-humanitarian-response-plan-draft https://reliefweb.int/report/zimbabwe/zimbabwe-humanitarian-response-plan-2020-march-2020 2) HRPs 2016 & 2021  <https://www.humanitarianresponse.info/en/operations/zimbabwe/document/zimbabwe-humanitarian-response-plan-2016>  <https://www.humanitarianresponse.info/en/operations/southern-eastern-africa/document/report-riasco-action-plan-el-ni%C3%B1o-induced-drought> 2016-2017  <https://www.humanitarianresponse.info/en/operations/zimbabwe/document/zimbabwe-drought-humanitarian-response-plan-april-2016-march-2017> |
| Economic collapse in 2008 continuing economic crisis<https://www.acaps.org/country/zimbabwe/crisis/complex-crisis> | South and West | 2008 | None identified |
| **SMALL STATES** | | | |
| **COMOROS** |  |  |  |
|  |  |  | <https://www.humanitarianresponse.info/en/operations/comoros/document/comoros-early-recovery-plan-flooding-2012> |
| **KIRIBATI** |  |  |  |
|  |  |  | None identified |
| **MARSHALL ISLANDS** |  |  |  |
|  |  |  | <https://www.humanitarianresponse.info/en/operations/marshall-islands/document/marshall-islands-nap-disaster-risk-management-2008-2018>  <https://reliefweb.int/report/micronesia-federated-states/north-pacific-islands-crisis-response-plan-2021-2023> |
| **MICRONESIA, FED. STS.** |  |  |  |
|  |  |  | <https://reliefweb.int/report/micronesia-federated-states/north-pacific-islands-crisis-response-plan-2021-2023> |
| **SOLOMON ISLANDS** |  |  |  |
| Ethnic violence (1998-2003) |  | 2014 | <https://www.humanitarianresponse.info/en/operations/solomon-islands/document/honiara-and-guadalcanal-flash-floods-humantarian-action-plan> |
| **TIMOR-LESTE** |  |  |  |
|  |  |  | None identified |
|  |  |  |  |
| Tuvalu |  |  | <https://www.humanitarianresponse.info/fr/operations/tuvalu/document/tuvalu-national-strategic-action-plan-climate-chanage-and-disaster-risk> |
