# Supplementary material for: Self-care interventions for sexual and reproductive health in humanitarian and fragile settings: a scoping review
Source: BMC Health Serv Res. 2022 Jun 7;22:757. doi: 10.1186/s12913-022-07916-4 (PMC9172979; doi:10.1186/s12913-022-07916-4)
Supplement: Supplementary file 2 — Additional file 2. Database Searches. [file 12913_2022_7916_MOESM2_ESM.docx]

**Additional file 2. Database Searches**

| Database: EMBASE | |
| --- | --- |
| Search terms |  |
| \|  \| (self-care or home birth or home-based or home administration or self-test or self-inject or self-administration or self-manage or over the counter or advance supply).ab. \| \| --- \| --- \| | 69426 |
| \|  \| (disaster or crisis or humanitarian).ab. \| \| --- \| --- \| | 82533 |
| \|  \| (Sexual or Reproductive or Maternal or Pregnancy or postpartum hemorrhage or newborn or cord care or thermal care or breastfeeding or contraception or abortion or post abortion or Violence or HIV post-exposure prophylaxis or STI presumptive treatment or rape or sexually transmitted).ab. \| \| --- \| --- \| | 12119117 |
| 1 and 2 and 3 | 26 |
| Database: Maternity and Infant Care |  |
| AB self-care or AB home birth or AB home-based or AB home administration or AB self-test or AB self-inject or AB self-administration or AB self-manage or AB over the counter or advance supply | 25687 |
| AB disaster or AB crisis or AB humanitarian | 29048 |
| \|  \| AB Sexual or Reproductive or AB Maternal or AB Pregnancy or AB postpartum hemorrhage or AB newborn or AB cord care or AB thermal care or AB breastfeeding or AB contraception or AB abortion or AB post abortion or AB Violence or AB HIV post-exposure prophylaxis or AB STI presumptive treatment or AB rape or AB sexually transmitted \| \| --- \| --- \| | 30108 |
| 1 and 2 and 3 | 30 |
| Database: Web of Science |  |
| \|  \| (self-care or home birth or home-based or home administration or self-test or self-inject or self-administration or self-manage or over the counter or advance supply).ab. \| \| --- \| --- \| | 47303 |
| \| (disaster or crisis or humanitarian).ab. \| \| --- \| | 62837 |
| \|  \| (Sexual or Reproductive or Maternal or Pregnancy or postpartum hemorrhage or newborn or cord care or thermal care or breastfeeding or contraception or abortion or post abortion or Violence or HIV post-exposure prophylaxis or STI presumptive treatment or rape or sexually transmitted).ab. \| \| --- \| --- \| | 913907 |
| 1 and 2 and 3 | 30 |
| Database: PubMed |  |
| **((self-care[Title/Abstract] OR home birth[Title/Abstract] OR home-based[Title/Abstract] OR home administration[Title/Abstract] OR self-test[Title/Abstract] OR self-inject[Title/Abstract] OR self-administration[Title/Abstract] OR self-manage[Title/Abstract] OR over the counter[Title/Abstract] OR advance supply[Title/Abstract]) AND (Sexual[Title/Abstract] OR Reproductive[Title/Abstract] OR Maternal[Title/Abstract] OR Pregnancy[Title/Abstract] OR postpartum haemorrhage[Title/Abstract] OR new-born[Title/Abstract] OR cord care[Title/Abstract] OR thermal care[Title/Abstract] OR breastfeeding[Title/Abstract] OR contraception[Title/Abstract] OR abortion[Title/Abstract] OR post abortion[Title/Abstract] OR Violence[Title/Abstract] OR HIV post-exposure prophylaxis[Title/Abstract] OR STI presumptive treatment[Title/Abstract] OR rape[Title/Abstract] OR sexually transmitted[Title/Abstract])) AND (disaster[Title/Abstract] OR crisis[Title/Abstract] OR humanitarian[Title/Abstract])** | 26 |
| Database: Medline |  |
| (self-care or home birth or home-based or home administration or self-test or self-inject or self-administration or self-manage or over the counter or advance supply).ab. | 48899 |
| \|  \| (Sexual or Reproductive or Maternal or Pregnancy or postpartum hemorrhage or newborn or cord care or thermal care or breastfeeding or contraception or abortion or post abortion or Violence or HIV post-exposure prophylaxis or STI presumptive treatment or rape or sexually transmitted).ab. \| \| --- \| --- \| | 936067 |
| disaster or crisis or humanitarian | 66293 |
| 1 and 2 and 3 | 18 |
| Database: CINAL |  |
| (self-care or home birth or home-based or home administration or self-test or self-inject or self-administration or self-manage or over the counter or advance supply).ab. | 30,540 |
| Sexual or Reproductive or Maternal or Pregnancy or postpartum hemorrhage or newborn or cord care or thermal care or breastfeeding or contraception or abortion or post abortion or Violence or HIV post-exposure prophylaxis or STI presumptive treatment or rape or sexually transmitted | 20438 |
| disaster or crisis or humanitarian | 30540 |
| 1 and 2 and 3 | 16 |
|  |  |
| Total | 120 |
| Minus duplicates |  |
| Screened | 27 |
| Removed at screening | 2 |
| Included in the scoping review | 25 |
